# Sensory-Guided Isolation, Identification, and Active Site Calculation of Novel Umami Peptides from Ethanol Precipitation Fractions of Fermented Grain Wine (Huangjiu)

**DOI:** 10.3390/foods12183398

**Published:** 2023-09-11

**Authors:** Rui Chang, Zhilei Zhou, Yong Dong, Yuezheng Xu, Zhongwei Ji, Shuangping Liu, Jian Mao

**Affiliations:** 1 National Engineering Research Center of Cereal Fermentation and Food Biomanufacturing, School of Food Science and Technology, Jiangnan University, Wuxi 214122, China; 7200112052@stu.jiangnan.edu.cn (R.C.);; 2 Jiangnan University (Shaoxing) Industrial Technology Research Institute, Shaoxing 312000, China; 3 Jiangsu Provincial Engineering Research Center for Bioactive Product Processing, Jiangnan University, Wuxi 214122, China; 4 National Engineering Research Center for Huangjiu, Shaoxing 312000, China

**Keywords:** huangjiu, umami peptide, molecular docking, peptidomics, density functional theory

## Abstract

Huangjiu is rich in low-molecular-weight peptides and has an umami taste. In order for its umami peptides to be discovered, huangjiu was subjected to ultrafiltration, ethanol precipitation, and macroporous resin purification processes. The target fractions were gathered according to sensory evaluation. Subsequently, we used peptidomics to identify the sum of 4158 peptides in most umami fractions. Finally, six novel umami peptides (DTYNPR, TYNPR, SYNPR, RFRQGD, NFHHGD, and FHHGD) and five umami-enhancing peptides (TYNPR, SYNPR, NFHHGD, FHHGD, and TVDGPSH) were filtered via virtual screening, molecular docking, and sensory verification. Moreover, the structure–activity relationship was discussed using computational approaches. Docking analysis showed that all umami peptides tend to bind with T1R1 through hydrogen bonds and hydrophobic forces, which involve key residues HIS71, ASP147, ARG151, TYR220, SER276, and ALA302. The active site calculation revealed that the positions of the key umami residues D and R in the terminal may cause taste differences in identified peptides.

## 1. Introduction

Umami is the fifth basic taste. Many studies have shown that not only can tasting molecules such as amino acids and nucleotides cause an umami taste, but low-molecular-weight polypeptides can also provide an umami taste [1]. The umami threshold values of most identified umami peptides are even lower than those of umami aspartate and glutamic acid [2], indicating the umami potential of peptides. In 1978, Yamasaki and Maekawa first discovered the umami-tasting octapeptide KGDEESLA from beef papain hydrolyzate [3]. Decades later, about 301 umami peptides have been identified [4], ranging in length from 2 to 15 amino acids, and many are derived from fermented foods. As a kind of typical fermented food, some types of grain-fermented wine have an obvious umami taste and are abundant with peptides [5,6]. Umami peptides are important nonvolatile flavor components in fermented wines, and they can affect overall sensory perception through their intrinsic taste profile and flavor interaction with the matrix’s components. In addition, several earlier studies revealed the umami potential of peptides in fermented wine [7,8,9]. However, due to the complex matrix and extraction process, there is still little known about umami peptides in fermented grain wine.

In order to collect the taste peptide fractions in food, preparative or semipreparative high-performance liquid chromatography separation has frequently been used, but it is very time-consuming [10]. Guided by sensory evaluation, the separation process can be effective even with a macroporous resin medium [11,12]. For peptide identification, the traditional instrument gives limited results due to accuracy issues. Recently, widely used peptidomics technology based on nano-liquid chromatography with tandem mass spectrometry (Nano-HPLC-MS/MS) and advanced data collection modes have been able to achieve high-throughput peptide sequencing results in crude fractions. Dozens of novel umami peptides have been discovered via peptidomics in chicken [13], fish [14], and bacon [15], and even the bioactive peptides in fermented rice wines have been discovered [16]. Moreover, peptidomics coupled with a series of in silico methods (machine learning, virtual screening, and molecular docking) has been an effective strategy for umami peptide prediction [17]. As of now, this strategy has successfully screened 16 umami peptides from 508 peptides in clams [18], and narrowed 208 chicken soup peptides down to 20 target peptides [13]. Therefore, peptidomics and virtual screening can contribute to the exploration of novel umami peptides in fermented wines.

Huangjiu is a traditional Chinese fermented rice wine that has been consumed for over 2000 years and has a distinct umami taste. During the fermentation, the raw protein is degraded by microbial metabolism and produces a wealth of peptides [6]. The peptide content (1770 to 4210 mg/L) of huangjiu is significantly higher than that of Japanese rice wine, also known as sake (156 to 242 mg/L) [5]. To date, 19 umami-enhancing glutamine peptides have been identified in sake [19,20], but only one potential umami peptide, EEE, has been reported in huangjiu [21]. Hence, the umami peptides in huangjiu warrant further exploration.

This study aims to enrich, identify, and characterize the huangjiu umami peptides through a combined isolation and screening strategy. The target fractions were separated via ultrafiltration, ethanol precipitation, and macroporous resin, and then evaluated by sensory panelists. After peptidomics sequencing and virtual screening, the candidate peptides were further verified through a sensory evaluation test. Finally, the taste mechanism of the identified umami peptides was explored through molecular docking and theoretical active site calculations. These results will provide a reference for taste peptide identification in other fermented wines.

## 2. Materials and Methods

### 2.1. Materials and Chemicals

The 20 L mechanical craft semidry (total sugar ranged from 15 to 40 g/L) huangjiu used in this study was sourced from a local winery in Shaoxing, Zhejiang province, China. All analytically pure reagents used, including absolute ethanol, sodium hydroxide, hydrochloric acid, phenol, concentrated sulfuric acid, anhydrous glucose, Folin phenol, and gallic acid, were procured from Sinopharm Reagent Co., Ltd., Shanghai, China. For the sensory test, food-grade sodium chloride, citric acid, sucrose, quinine, glutathione, and monosodium glutamate (MSG) were purchased from Gino Biotech Co., Ltd., Quzhou, China. The purification equipment, specifically the membrane filtration module, was acquired from Shaoxing Haina Co., Ltd., Shaoxing, China.

### 2.2. Separation of Huangjiu Umami Peptide Fractions

**Figure 1:**
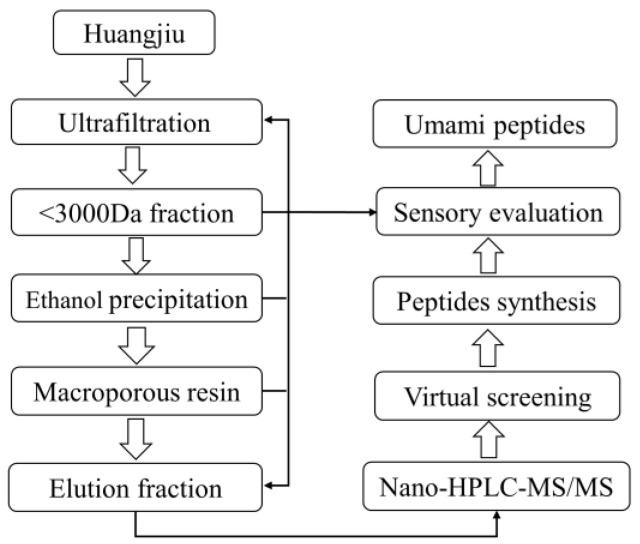
The flowchart of the huangjiu umami peptide separation and identification.

The target umami peptide fractions were obtained through sensory evaluation of the isolate separated via ultrafiltration, ethanol precipitation, and macroporous resin processes (Figure 1). First, the raw huangjiu was subjected to centrifuge pretreatment (8000 rpm, 20 min, 4 °C) to remove insoluble impurities, and then it was passed through an ultrafiltration membrane with a molecular weight cut-off of 3000 Da (Suez, Wuxi, China). The permeate fraction (HJUF) with peptide molecular weight lower than 3000 Da was concentrated using rotary evaporation (IKA RV8, Staufen, Germany) at 50 °C and a vacuum of <0.098 MPa, followed by freeze-drying. The HJUF, with a peptide concentration of 40 mg/mL, was sequentially subjected to 60%, 75%, and 90% ethanol gradient precipitation (4 °C, 12 h). The precipitates were collected after centrifugation and named 60% ETP, 75% ETP, and 90% ETP, respectively. The final supernatant was termed 90% EST. The concentrations of peptides, total sugars, and total phenols in all fractions were quantified using the Pierce™ BCA Peptide Quantification Kit (Thermo Fisher Scientific, San Jose, CA, USA), phenol sulfate colorimetry, and Folin phenol colorimetry, respectively. The peptide content (g/100 g) was calculated as its purity in the sum of the three above components.

The preliminary fractions 60% ETP, 75% ETP, and 90% ETP were dissolved in pure water at a peptide concentration of 20 mg/mL, then loaded (15 mL) onto a chromatography column (Φ2.6 × 18 cm) equipped with nonpolar XAD16 macroporous resin (Shanghai Yuanye Biology, Shanghai, China). After reaching adsorption equilibrium, the column was sequentially eluted with 0%, 20%, 40%, and 60% ethanol at a flow rate of 3 mL/min. The elution process was monitored at 220 nm ultraviolet absorption value. The eluted samples corresponding to the same absorption peaks were collected together, concentrated, and then freeze-dried for subsequent sensory analysis.

### 2.3. Identification of Peptides via Nano-HPLC-MS/MS

The peptide fraction with the highest sensory umami intensity was identified using Nano-HPLC-MS/MS. The samples were desalted using Pierce C18 Spin Tips and then redissolved in solvent A (0.1% formic acid in water), followed by analysis using Q-Exactive Plus coupled to an EASY-nanoLC 1200 system (Thermo Fisher Scientific, San Jose, CA, USA). The sample (6 µL) was loaded onto an analytical column of 25 cm with 75 μm inner diameter and 1.9 μm resin (Dr Maisch) at a flow rate of 300 nL/min, then separated at 2% buffer B (80% ACN with 0.1% FA) with a 60 min gradient at the start, increasing stepwise to 35% within 47 min and then to 100% in 1 min, which was then maintained for 12 min. The column temperature was 40 °C and the electrospray voltage was set to 2 kV. The mass spectrometer was run under the data-dependent acquisition (DDA) mode. The full-scan MS spectra (m/z 200–1800) survey was conducted with a resolution of 7 × 10^4^. The automatic gain control (AGC) target was 3 × 10^6^, and the maximum injection time was 50 ms. Next, the precursor ions were selected in the collision cell for fragmentation via higher-energy collision dissociation (HCD). The MS/MS resolution was set to 1.75 × 10^4^, and the AGC target was 1 × 10^5^, the maximum injection time was 45 ms, and the dynamic exclusion was 30 s.

Data were processed using PEAKS Studio version 10.6 (Bioinformatics Solutions Inc., Waterloo, ON, Canada) and then matched with the proteomes of Oryza sativa subsp. Japonica, Triticum aestivum, and Saccharomyces cerevisiae (strain ATCC 204508/S288c) in the UniProt database. No digestion enzyme was selected. The peptides with a −logP score of ≥20 were filtered.

### 2.4. Virtual Screening and Molecular Docking

Considering the length of most reported umami peptides, the identified peptides with lengths no longer than ten were kept for further analysis. Machine learning models embedded in online discrimination tools were used to predict the umami potential of the peptide sequences. Potential umami peptides were retained through BIOPEP-UWM [22] (https://biochemia.uwm.edu.pl/biopep-uwm/ accessed on 15 June 2023), UMPred-FRL [23] (http://pmlabstack.pythonanywhere.com/UMPred-FRL accessed on 15 June 2023), and Umami_YYDS [4] (http://www.tastepeptides-meta.com/cal, accessed on 15 June 2023). The filtered peptides were kept for screening.

Molecular docking of filtered umami peptides was conducted with the T1R1/T1R3 homology model, which was built at SWISS-MODEL (https://swissmodel.expasy.org/, accessed on 16 June 2023). The sweet receptor (PDB code: 5X2M) was chosen as a template, and the sequences of human umami receptors Q7RTX1 (TSIR1) and Q7RTX0 (TSIR3) were retrieved from the UniProt database [24]. The model stereochemistry was assessed with the MolProbity server (http://molprobity.biochem.duke.edu/, accessed on 16 June 2023). The peptide structures were generated with Chemoffice software after MMFF94 force field energy optimization. Docking was performed with Auto Dock Vina1.1.2. The docking active sites of the receptor were determined through alignment with the original ligand of the template protein, T1R3 site x, y, z = 51.31, 40.37, 12.71 and T1R1 site x, y, z = 39.05, 25.33, 40.88, with the box region being 25 × 25 × 25 Å. The ratio of hydrophobic, acidic, basic, bitter, and sweet amino acids in all candidate synthetic peptide sequences was counted, and the taste activity profile was also calculated using BIOPEP-UWM sensory analysis.

### 2.5. Solid-Phase Synthesis of Umami Peptides

The ten candidate umami peptides screened from docking score were synthesized with the solid-phase synthesis method from Shanghai Gill Biochemical Co., Ltd. (Shanghai, China), with purity not lower than 95%.

### 2.6. Sensory Evaluation

#### 2.6.1. Descriptive Sensory Analysis

Referring to the quantitative descriptive analysis method reported by Zhuang et al. [11] with slight modifications, the sensory evaluation was conducted inside a room at a temperature of 24 °C, in accordance with ISO-8586-1, 2012. Ten sensory evaluation panelists (five men and five women, aged from 25 to 30) who have no history of known taste impairment or disorders were recruited from Jiangnan University. The panelists could accurately identify and describe the characteristic differences in taste standard solutions, including sour, sweet, bitter, salty, umami, and kokumi, after undergoing sensory training. All panelists gave their informed consent before every sensory session, which was approved by the Jiangnan University Medical Ethics Committee (JNU20230601IRB21). The panelists were asked to describe the samples’ taste and assign intensity scores ranging from 0 to 9 points, with 0 for pure water and 5 representing standard solutions. The standard taste solutions for sour, sweet, bitter, salty, umami, and kokumi were 0.08% citric acid, 1% sucrose, 0.08% quinine, 0.35% sodium chloride, 0.35% monosodium glutamate (MSG), and 0.15% glutathione, respectively. All of the peptide fractions obtained via ultrafiltration, ethanol precipitation, and resin separation were dissolved in water at a concentration of 10 mg/mL. The candidate synthetic peptide was dissolved in water at a concentration of 4 mg/mL. The panel members sipped each sample for 10 s prior to spitting it out, washed their mouths with pure water, and rested for 3 min to eliminate fatigue. The final sensory intensity score was the average of all panelists’ scores.

#### 2.6.2. Taste Thresholds of Synthetic Peptides

According to the methods of Liu et al. [25] with slight modifications, the synthetic peptides were diluted with water at a 1:1 (*v*/*v*) ratio, and judgment started as the concentration increased. The possible concentration range of threshold was preliminarily determined based on the descriptive sensory trial. Namely, 0.125 mg/mL was used as the initial evaluation concentration, followed by 0.25, 0.5, 1, and 2 mg/mL. Triangle tests were performed on one diluted peptide sample group and two water blank control groups. The individual thresholds of all the panelists were averaged to determine the overall threshold.

#### 2.6.3. Umami Enhancement Effects of Synthetic Peptides

The peptides were dissolved in 0.35% MSG solutions to reach 1 mg/mL and then given to the panelists for umami intensity evaluation, separately, for which the intensity score range was 0 to 9. The blank solution 0.35% MSG was scored as 5. For the thresholds of umami enhancement, a comparative taste dilution analysis was adopted [13].

### 2.7. Theoretical Calculation of Active Sites

All ten candidate peptides’ active sites were calculated. Geometry optimization of peptide structure was performed at the B3LYP level, considering the D3 dispersion correction for weak interaction. Given that the construction of the Pople basis set is not ideal, the higher-precision def series 3-zeta basis set TVZP was used. In addition, the environment was applied with gas-phase and implicit solvation (water with SMD model). All calculations were performed using the ORCA package (version 5.03), and the hybrid functional RI acceleration technology was fully utilized to reduce calculation time. Molecular orbital analysis (HOMO and LUMO) was conducted with the Multiwfn program (version 3.7) [26].

### 2.8. Data Analysis

All assays were conducted in triplicate. Data analysis of variance (ANOVA) and Duncan’s test were performed on pairs using SPSS (version 23.0, SPSS Inc., Chicago, IL, USA). Results were deemed statistically significant at *p* < 0.05. Data visualization was carried out using Origin software (Pro 2016, Origin Lab Corp., Northampton, MA, USA). The interactions between the peptides and the receptor during docking were visualized using Ligplot+, and the interaction bonds’ heatmap was drawn using Hiplot (https://hiplot.com.cn/, accessed on 1 August 2023).

## 3. Results and Discussion

### 3.1. Sensory-Guided Separation of Huangjiu Peptides

#### 3.1.1. Ultrafiltration and Ethanol Precipitation Fractions

**Figure 2:**
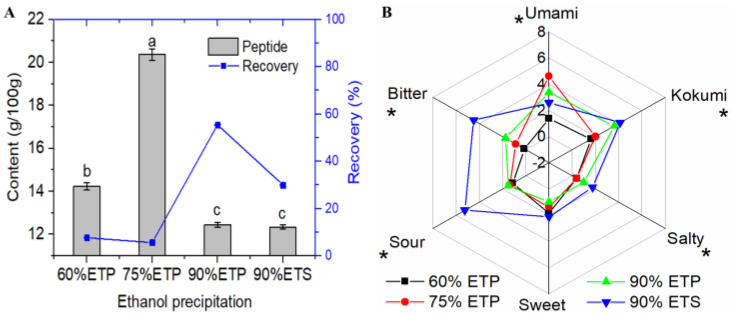
Sensory characteristics of precipitated huangjiu umami peptide fractions ((**A**): recovery rate and peptide content, (**B**): sensory intensity of peptide fractions). Note that letters in the same column and asterisks in sensory radar chart represent significant differences (*p* < 0.05).

Compared with the raw amount (7.68 ± 0.36 g/100 g), the peptide content of the permeate fraction (HJUF) was increased to 12.88 ± 0.19 g/100 g after ultrafiltration. However, the umami intensity of HJUF did not improve very much, indicating that the umami fraction needed further enrichment (Figure 2B). The peptide content and recovery rate of ethanol precipitation (60% ETP, 75% ETP, 90% ETP) and the supernatant fraction (90% EST) are shown in Figure 2A. The results showed that a total of 68.81% of the peptides were recovered through ethanol precipitation, suggesting that most huangjiu peptides have a certain degree of hydrophobicity. Hydrophobicity is an important characteristic of umami peptides [27]. In addition, the precipitation process increased the peptide content of the 75% ETP fractions (20.36 ± 0.26 g/100 g), as well as the 60% ETP fractions (14.22 ± 0.56 g/100 g). Although the peptide purity of all precipitated fractions was not very high, the sensory panelists could still distinguish the taste difference. The sensory results showed that 60% ETP was tasteless, and 75% ETP had the highest umami intensity (score: 4.6), followed by 90% ETP (score: 3.38). Meanwhile, the 90% EST fraction had a strong bitter taste and high acidity.

#### 3.1.2. Macroporous Resin Separation Fractions

**Figure 3:**
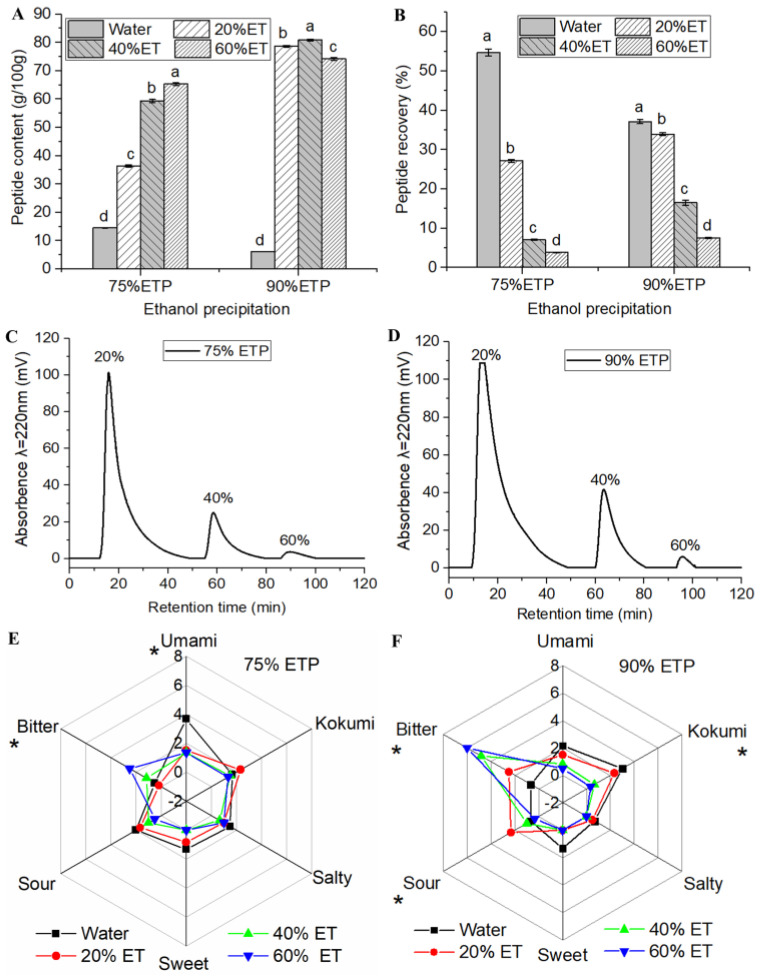
Sensory characteristics of resin−purified huangjiu umami peptide fractions ((**A**): peptide content, (**B**): recovery rate, (**C**,**D**): elution response curve, (**E**,**F**): sensory intensity of each elution fraction). The asterisks in sensory radar chart represent significant differences (*p* < 0.05). Different letters above bars indicate significant differences at the 0.05 level.

Resin was used for further enrichment. Macroporous resin, with its high loading capacity, safe eluent, and simple purification steps, has been applied for taste peptide fraction enrichment in soy sauce [28,29] and bean paste [30]. The nonpolar resin XAD-16 has a large specific surface area (800 m^2^/g) and a small pore size (0.7 nm), which have led to it being commonly used in complex food material matrices. In particular, it performed well in terms of grain protein hydrolyzate peptide enrichment [31], and was thus applied to fermented grain wine, or huangjiu. In this study, the highest umami fractions, 75% ETP and 90% ETP, were kept separated via macroporous resin separation, and different hydrophobicity samples were obtained through ethanol gradient elution (Figure 3C,D).

With elution, the peptide content of the collected samples gradually increased (Figure 3A). Most peptides in 75% ETP and 90% ETP were eluted with water (0% ethanol), with recovery rates of 54.71 ± 0.93% and 37.15 ± 0.52%, respectively (Figure 3B). Notably, the water-eluted fractions had higher umami intensity than the 20~60% ethanol-eluted samples (Figure 3E,F), even though the peptide content was much lower (Figure 3A). Meanwhile, the 20–60% ethanol-eluted fractions had a taste dominated by bitterness. Similarly, in the soy sauce umami peptide macroporous resin separation study, the author also observed that the water-eluted resin fractions had a higher umami intensity than other fractions [11]. Comparing the water-eluted umami fractions of 75% ETP and 90% ETP, the umami intensity of the former (3.67) was almost twice that of the latter. Furthermore, the water-eluted fractions of 75% ETP had a strong sour taste, which may be due to the presence of more acidic umami amino acid residues in the peptides. For instance, the umami taste of Korean soybean paste [32] and stinky mandarin fish [14] peptides was correlated to a high proportion of acidic amino acids (glutamic acid and aspartic acid). Unlike soy sauce and soybean paste, stinky mandarin fish is a fermented meat product, but its umami peptide composition also relies on hydrophilic amino acids. This suggests that for umami peptides in fermented foods, moderate hydrophilicity is a necessary key factor. Therefore, the huangjiu umami peptides may be concentrated in the 75% water-eluted resin fractions.

### 3.2. Virtual Screening of Umami Peptides

Nano-HPLC-MS/MS identified 4158 peptides from the 75% ETP water-eluted fractions. There are 22 peptides with the greatest potential umami taste according to the machine learning prediction tools (BIOPEP-UWM, Umami_PREL, Umami_YYDS). The MolProbity Ramachandran plot (Figure S1) showed that the stereochemistry of the built receptor model residues was reasonable, with 93.7% in the favored area and 98.7% in the allowed area, similar to the results of Cheng et al. [33]. From the docking results (Table S1), ten peptides with intensities larger than 5 × 10^6^ and binding energies lower than −8.9 kcal/mol were selected for solid-phase synthesis and sensory evaluation. The protein accession matching analysis showed that DTYNPR, TYNPR, SYNPR, FRDEHQ, FRDEH, and RFRQGD were derived from the rice glutenin subunit. Among them, DTYNPR was reported as a lipolysis inhibitory peptide [34]. TYNPK came from the wheat helicase MAGATAMA 3. NFHHGD, FHHGD, and TVDGPSH were mainly released from the yeasts Enolase 2 and Glyceraldehyde-3-phosphate dehydrogenase 1. These findings demonstrated that enzymatic hydrolysis of raw proteins (rice, wheat, yeast, etc.) and yeast autolysis are important sources of huangjiu umami peptides [5].

### 3.3. Synthetic Peptide Umami Characteristics and Composition Analysis

**Figure 4:**
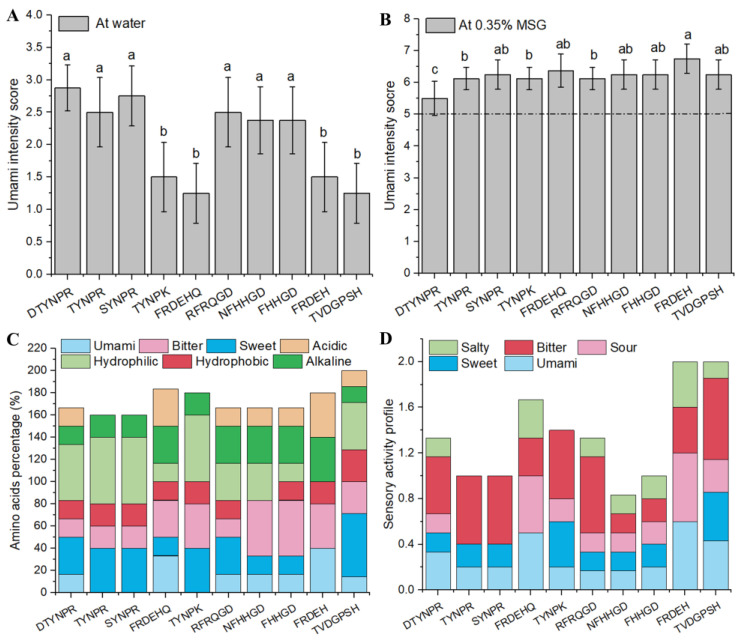
Sensory and sequence features of synthetic umami peptides ((**A**): umami intensity in water, (**B**): umami-enhancing effect with 0.35% MSG, (**C**): amino acid composition, (**D**): taste activity profile). Different letters in the same column represent significant differences (*p* < 0.05).

Sensory evaluation confirmed that DTYNPR, TYNPR, SYNPR, RFRQGD, NFHHGD, and FHHGD had a prominent umami taste (Figure 4A). The taste thresholds of these peptides in water were 1.63 ± 0.60, 2.11 ± 0.80, 2.36 ± 0.84, 2.41 ± 1.11, 2.75 ± 1.28, and 2.86 ± 0.75 mmol/L, respectively (Table 1). Although the thresholds of these peptides in water were higher than that of MSG (1.6 mmol/L), most of the peptides had umami-enhancing effects in 0.35% MSG solutions, and the threshold values ranged from 0.44 ± 0.15 to 0.65 ± 0.19 mmol/L. Notably, the weakly umami FRDEHQ had the best umami-enhancing effect in the 0.35% MSG solution, improving umami intensity up to 6.75 at 1 mg/mL, with a corresponding threshold value of 0.44 ± 0.15 mmol/L. Similarly, the TYNPK, FRDEH, and TVDGPSH peptides also significantly increased umami intensity in 0.35% MSG (Figure 4B). This enhancement of umami may be due to the synergistic effect of MSG with umami peptides. In a relevant study [35], the results showed that the addition of MSG increased the size of the receptor binding cavity and allowed peptides to bind to more key residues, thus inducing a stronger umami flavor. Therefore, the docking screening effectively identified peptides associated with umami tastes, but further analysis is needed to understand the differences in detail.

The amino acid composition analysis revealed that most peptides shared umami residues D or E (Figure 4C). However, the taste activity profile indicated that umami amino acids are not necessary conditions for peptides’ umami taste potential (Figure 4D) [36]. TYNPR and SYNPR lack umami amino acids but have an umami taste, which can be attributed to the umami dipeptide fragment NP [37], the C-terminal amino acids R [38], and the geometry of binding with the T1R1-T1R3 receptor’s key residues. Umami amino acids are also acidic amino acids. Due to the peptides FRDEHQ and FRDEH both having the sour character of residues D and E and the fragment DE, they provide a stronger sour sensory taste than others. This aligns with the acidic dipeptides DD, DE, EE, and EEE found in soy sauce [39].

In addition, basic (R, K) and bitter amino acids (F, H, Y) also appear frequently. R and K have often been found in reported umami peptides, such as clam water extract umami peptides [40]. All of the peptides with a high taste activity profile for bitterness were not too bitter, this may be due to the masking effect of umami, sweetness, and basic fragments on bitterness [41]. Like TVDGPSH, the bitterness profile was high (0.71), but the umami fragments (VD, D) and sweet amino acids (V, G, P) contribute to the total profile score up to 0.8572 (Figure 3D), and the sensory evaluation showed that the bitterness was very weak. In a metabolomics study of taste components in pufferfish [42] and salted duck eggs [43], the author found that some non-umami amino acids can enhance umami intensity. This suggests that the synergistic effect of sweet and umami amino acids is beneficial for reducing bitterness. Similarly, the results obtained by Wang et al. also showed that sweet amino acids (A, S, G, etc.) and acidic residues D/E can significantly improve umami taste [44]. Therefore, amino acid composition is closely related to the umami taste of peptides.

### 3.4. Molecular Docking Interactions of Peptides with T1R1-T1R3

**Figure 5:**
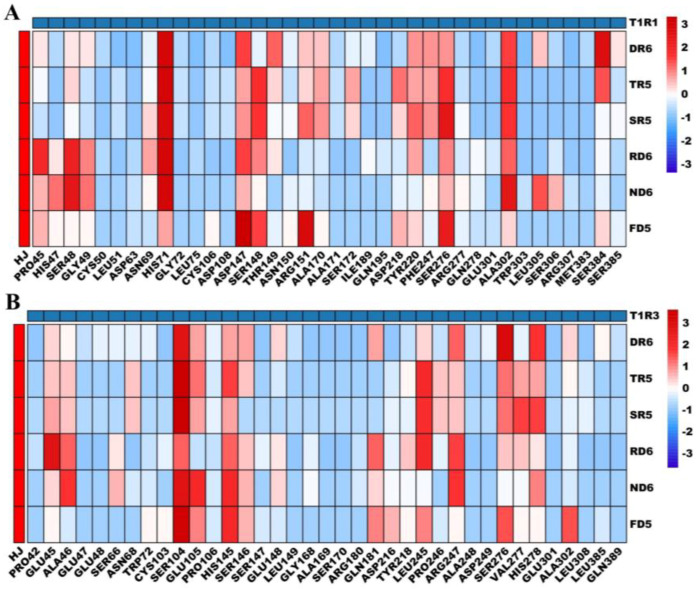
The interaction bond heatmap between umami peptides and T1R1−T1R3 receptors ((**A**) for T1R1 and (**B**) for T1R3. DR6, TR5, SR5, RD6, ND6, and FD5 are short for DTYNPR, TYNPR, SYNPR, RFRQGD, NFHHGD, and FHHGD, respectively).

In order to investigate the interaction between peptides and T1R1-T1R3 receptors, a docking analysis was performed. The 2D residue interactions are shown in Figure S2 and the corresponding contact bond number heatmap is depicted in Figure 5.

#### 3.4.1. Molecular Docking Interactions of Peptides with T1R1

As seen from the docking energy results in Table S1, the six umami peptides tended to interact with T1R1 due to its lower binding energy (−9.6 to −11.0 kcal/mol) compared with T1R3 (−8.9 to −10.1 kcal/mol). This was consistent with the reported studies showing that T1R1 was the main umami peptide binding domain [27,44]. And the affinity of most of the umami peptides, DTYNPR, TYNPR, and SYNPR, to T1R1 was also lower than that of RFRQGD, NFHHGD, and FHHGD. This indicated that binding affinity can be used for evaluating peptides. The results of the residue interaction (Figure S2) revealed that these peptides formed 14, 15, 16, 16, 11, and 14 hydrogen bonds, respectively, accounting for 10.18% to 20.25% of the total contact bonds, while the hydrophobic bonds occupied 86, 69, 63, 105, 97, and 95, respectively. Therefore, hydrophobic forces were a crucial factor for peptide–receptor binding. This process involved a number of high contact frequency residues including SER48, HIS71, ASP147, SER148, ARG151, TYR220, PHE247, SER276, ALA302, and SER384. Among these, SER48, ARG151, TYR220, and SER276 were key residues for the umami molecules GMP and IMP and the beef peptide KGDESSLA with T1R1 [24]. The residues HIS71, ASP147, and ALA302 also contributed the most to the chicken soup and pufferfish umami peptides with T1R1 [13,45]. In addition, when compared with RFRQGD, NFHHGD, and FHHGD, three other umami peptides (DTYNPR, TYNPR, and SYNPR) can still bind with some residues (THR149, ALA170, and SER172) that also play important roles for monosodium glutamate and sodium succinate [24].

#### 3.4.2. Molecular Docking Interactions of Peptides with T1R3

For T1R3, the hydrogen bond numbers of the above six peptides dropped to 10, 6, 6, 9, 7, and 5, respectively (Figure S2). Meanwhile, the hydrophobic interactions were also reduced to 90, 62, 58, 82, 72, and 58, respectively, amounting to 90.11% to 92.06% of the total contact bonds. From the heatmap results, all peptides mostly shared the residues GLU45, SER104, HIS145, LEU245, ARG247, SER276, VAL277, and HIS278.

The residues GLU45, HIS145, VAL277, and HIS278 were also crucial for the chicken breast umami peptides GIQKELQF, FTERVQ, and AEINKILGN binding to T1R3 [46]. In the docking results for porcine bone soup [47] and fermented fish (Chouguiyu) umami peptides [14] with T1R3, the residues SER104, HIS145, and SER276 were important. Beyond that, the residues GLU45, SER104, LEU245, and HIS278 may have contributed to the umami-enhancing effect, as suggested by the results of the umami-enhancing peptide RFPHADF-ARP docking with T1R3 [48]. This suggests that T1R3 was also necessary in umami recognition. Moreover, it was noted that DTYNPR, TYNPR, and SYNPR interacted more with key residues such as ASN68, LEU245, SER276, VAL277, and HIS278 than others (RFRQGD, NFHHGD, and FHHGD). Furthermore, TYNPR and SNPR interacted with exactly the same residues with T1R3, which may explain the similar umami intensities.

### 3.5. Theoretical Calculation of Umami Peptides’ Active Sites

**Figure 6:**
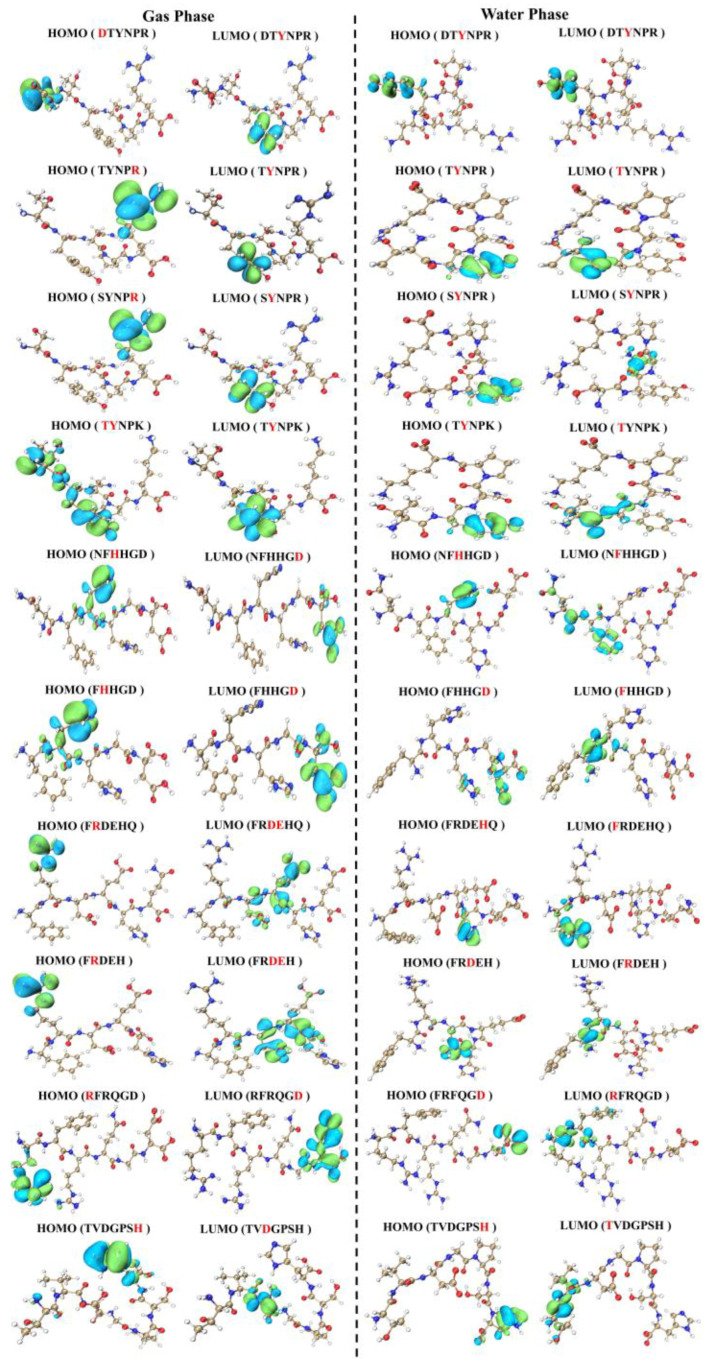
The calculated active sites of umami peptides (active sites are colored red in sequence, and the positive and negative phases of the orbital wave function are shown in green and blue, respectively).

The ten identified peptides above (Table 1) have similar binding energy ranges but differ significantly in umami intensity, indicating that the affinity energy is just one of the necessary conditions for umami peptides. To further investigate the differences between these umami peptides, active site calculations were performed in both the gas and water phases, and the results are shown in Figure 6. In general, the active sites of molecules are regions that are prone to gaining and losing electrons, and the electron density distribution in these regions is different from that in others. Density functional theory (DFT) has been widely used to describe the structure of atoms, molecules, crystals, etc., based on the electron density distribution, and the molecular orbital is directly related to the electron density. In general, the active sites can be reflected by the highest occupied molecular orbital (HOMO) and the lowest unoccupied molecular orbital (LUMO), which refer to the regions that tend to donate and accept electrons, respectively [49].

The results indicate (Figure 6) that most of the positions of HOMO and LUMO on huangjiu umami peptides in the gas and water phases were not the same. This revealed that either HOMO or LUMO could be the active sites, and the solvent environment had a great influence on them. According to Wang et al.’s study [44], the residues D, E, R, K, and H in medium-length (4–7) umami peptides are prone to being the active sites. Our results show that the terminal residues (D or R) in the DTYNPR, TYNPR, SYNPR, FHHGD, NFHHGD, and RFRQGD peptides tend to be active sites in the gas phase. This confirmed the positional distribution of umami peptide active sites proposed by Wang et al. However, the results were different in the water phase; only FHHGD and RFRQGD still had the active sites D and R, which may be related to the protonation or deprotonation of peptide amino and carboxyl groups in the water environment. Since the protonation process of peptides can be influenced by varying pH conditions, many studies have calculated the active sites of small peptides under gas-phase conditions for convenient comparison [44,48,50].

As we take the sensory evaluation and DFT calculation results together, it appears that the position and types of active sites (D, E, R, and K) in the ten peptides may have the most impact on the umami taste, which is consistent with the results of Wang et al.’s study [44]. DTYNPR, TYNPR, and SYNPR all had the terminal residues R or D, and they act as active sites in the gas phase, but the C-terminal residue K at TYNPK was not one of the active sites (Y and T), which could have caused the weaker umami intensity of TYNPK. Furthermore, it is known that residues D and E at the length of 4–7 umami peptides are most involved in docking with T1R1 receptors [40]. The peptides FRDEHQ, FRDEH, and TVDGPSH, which have a weaker umami taste, had the key active sites of D or E, but these sites were also not located at the terminal position. When key active sites (D, E, and R) are in the terminal position, it may be beneficial to reduce the steric hindrance when interacting with the T1R1 receptor pocket. In addition, for peptides with multiple active sites (D, E, H, and R), the umami intensity may be affected by whether the key binding residues D or E are preferred at the terminal position. This inference was confirmed with the ten umami peptides identified above, and this offered a possible explanation for the ten peptides’ umami taste intensity. These results further proved the relationship between the peptide surface charge and taste activity [51], because the most active sites (charged amino acids or groups) tend to change position according to different electron densities. In a recent study, when the terminal residues of the bitter peptide RFPHADF were modified with glucose to increase electron density through the Maillard reaction, the original bitter peptide could be transformed into a non-bitter umami-enhancing peptide [48]. Therefore, the above analysis demonstrated that the position of key active sites in small peptides has a significant effect on the overall taste.

## 4. Conclusions

In this study, six novel umami and five umami-enhancing peptides derived from huangjiu were identified through multistep purification, virtual screening, and sensory evaluation. All identified umami peptides could bind to the T1R1-T1R3 receptor’s key residues through hydrogen bonds and van der Waals forces. Furthermore, the active site calculations revealed that the key umami residues (D and R), whether at the sequence’s terminal position or not, had a significant impact on the taste features of the above huangjiu umami peptides. In the future, studying peptide stability and regulation during the brewing process will provide a better understanding of the fermented grain wine’s taste. Overall, peptitomics combined with sensory-guided isolation could contribute to the discovery of taste peptides in other fermented wines and complex food matrices.

## Figures and Tables

**Table 1:** Umami thresholds of target synthetic peptides.

Peptide	Sensory Description	Umami Threshold (mol/L)		Protein
		in Water in MSG		Accession
DTYNPR	umami, slightly sweet, sour	1.63 ± 0.60 b	0.81 ± 0.28 a	P07728
TYNPR	umami, slightly bitter	2.11 ± 0.80 ab	0.63 ± 0.18 b	P14614
SYNPR	umami, slightly bitter	2.36 ± 0.84 ab	0.64 ± 0.19 ab	Q02897
FRDEHQ	weakly umami, sour	3.02 ± 0.56 a	0.65 ± 0.19 ab	P14614
TYNPK	weakly umami, slightly bitter	2.41 ± 1.11 ab	0.53 ± 0.13 b	A0A3B6AZR2
RFRQGD	umami, sour	2.41 ± 0.45 ab	0.53 ± 0.16 b	P07728
NFHHGD	umami, weakly sour	2.75 ± 1.28 a	0.52 ± 0.17 b	P00925
FHHGD	umami, weakly sour	2.86 ± 0.75 a	0.61 ± 0.20 ab	P00925
FRDEH	weakly umami, sour	3.02 ± 1.18 a	0.44 ± 0.15 b	P14614
TVDGPSH	weakly umami, weakly sour	3.16 ± 0.99 a	0.61 ± 0.16 ab	P00360

Results are mean ± SD (n = 3); different letters in the same column represent significant differences (*p* < 0.05).

## Data Availability

Data will be made available on request.

## Supplementary Materials

The following supporting information can be downloaded at https://www.mdpi.com/article/10.3390/foods12183398/s1: Table S1: Umami prediction and docking screening results of 75% ETP water-eluted fraction peptides with macroporous resin; Figure S1: Ramachandran plot of the modeled umami receptor T1R1/T1R3; Figure S2: The docking residue interaction mode between umami peptides and T1R1-T1R3 receptor.

## References

[1] Kurihara K. (2009). Glutamate: From discovery as a food flavor to role as a basic taste (umami). Am. J. Clin. Nutr..

[2] Huang Z., Feng Y., Zeng J., Zhao M. (2023). Six categories of amino acid derivatives with potential taste contributions: A review of studies on soy sauce. Crit. Rev. Food Sci. Nutr..

[3] Yamasaki Y., Maekawa K. (1978). A peptide with delicious taste. Agric. Biol. Chem..

[4] Cui Z., Zhang Z., Zhou T., Zhou X., Zhang Y., Meng H., Wang W., Liu Y. (2023). A TastePeptides-Meta system including an umami/bitter classification model Umami_YYDS, a TastePeptidesDB database and an open-source package Auto_Taste_ML. Food Chem..

[5] Zhou M., Bu T., Zheng J., Liu L., Yu S., Li S., Wu J. (2021). Peptides in brewed wines: Formation, structure, and function. J. Agric. Food Chem..

[6] Shi Y., Feng R., Mao J., Liu S., Zhou Z., Ji Z., Chen S., Mao J. (2021). Structural characterization of peptides from huangjiu and their regulation of hepatic steatosis and gut microbiota dysbiosis in hyperlipidemia mice. Front. Pharmacol..

[7] Schmidt C.V., Olsen K., Mouritsen O.G. (2021). Umami potential of fermented beverages: Sake, wine, champagne, and beer. Food Chem..

[8] Klosse P. (2013). Umami in wine. Res. Hosp. Manag..

[9] Yamamoto T., Watanabe U., Fujimoto M., Sako N. (2009). Taste preference and nerve response to 5′-inosine monophosphate are enhanced by glutathione in mice. Chem. Senses.

[10] Ding Y., Li X., Kan J. (2017). Isolation and identification of flavor peptides from douchi (traditional Chinese soybean food). Int. J. Food Prop..

[11] Zhuang M., Zhao M., Lin L., Dong Y., Chen H., Feng M., Sun-Waterhouse D., Su G. (2016). Macroporous resin purification of peptides with umami taste from soy sauce. Food Chem..

[12] Fan S.H., Liu T.T., Wan P., Zhu Q., Xia N., Wang Q.Z., Chen D.W. (2021). Enrichment of the umami-taste-active amino acids and peptides from crab sauce using ethanol precipitation and anion-exchange resin. J. Food Process. Preserv..

[13] Zhang J., Zhang J., Liang L., Sun B., Zhang Y. (2023). Identification and virtual screening of novel umami peptides from chicken soup by molecular docking. Food Chem..

[14] Yang D., Li C., Li L., Chen S., Hu X., Xiang H. (2022). Taste mechanism of umami peptides from Chinese traditional fermented fish (Chouguiyu) based on molecular docking using umami receptor T1R1/T1R3. Food Chem..

[15] Zhang J., Toldrá F., Zhang W., Yin Y., Zhu Z. (2023). Study on the effects and mechanisms of ultrasound on the peptide profile and taste of unsmoked bacon using peptidomics and bioinformatics. Food Chem..

[16] Zheng X., Chi H., Ma S., Zhao L., Cai S. (2023). Identification of novel α-glucosidase inhibitory peptides in rice wine and their antioxidant activities using in silico and in vitro analyses. LWT.

[17] Qi L., Gao X., Pan D., Sun Y., Cai Z., Xiong Y., Dang Y. (2022). Research progress in the screening and evaluation of umami peptides. Compr. Rev. Food Sci. Food Saf..

[18] Zhang T., Hua Y., Zhou C., Xiong Y., Pan D., Liu Z., Dang Y. (2022). Umami peptides screened based on peptidomics and virtual screening from *Ruditapes philippinarum* and *Mactra veneriformis* clams. Food Chem..

[19] Kiyono T., Hirooka K., Yamamoto Y., Kuniishi S., Ohtsuka M., Kimura S., Park E.Y., Nakamura Y., Sato K. (2013). Identification of pyroglutamyl peptides in Japanese rice wine (sake): Presence of hepatoprotective pyroGlu-Leu. J. Agric. Food Chem..

[20] Hashizume K., Ito T., Nagae Y., Tokiwano T. (2019). Quantitation and sensory properties of three newly identified pyroglutamyl oligopeptides in sake. Biosci. Biotechnol. Biochem..

[21] Han F.L., Xu Y. (2011). Identification of low molecular weight peptides in Chinese rice wine (Huang Jiu) by UPLC-ESI-MS/MS. J. Inst. Brew..

[22] Minkiewicz P., Iwaniak A., Darewicz M. (2019). BIOPEP-UWM database of bioactive peptides: Current opportunities. Int. J. Mol. Sci..

[23] Charoenkwan P., Nantasenamat C., Hasan M.M., Moni M.A., Manavalan B., Shoombuatong W. (2021). UMPred-FRL: A new approach for accurate prediction of umami peptides using feature representation learning. Int. J. Mol. Sci..

[24] Liu H., Da L.-T., Liu Y. (2019). Understanding the molecular mechanism of umami recognition by T1R1-T1R3 using molecular dynamics simulations. Biochem. Biophys. Res. Commun..

[25] Liu Z., Zhu Y., Wang W., Zhou X., Chen G., Liu Y. (2020). Seven novel umami peptides from Takifugu rubripes and their taste characteristics. Food Chem..

[26] Lu T., Chen F. (2012). Multiwfn: A multifunctional wavefunction analyzer. J. Comput. Chem..

[27] Zhang C., Miao Y., Feng Y., Wang J., Tian Z., Dong J., Gao B., Zhang L. (2022). Umami polypeptide detection system targeting the human T1R1 receptor and its taste-presenting mechanism. Biomaterials.

[28] Zhu X., Sun-Waterhouse D., Chen J., Cui C., Wang W. (2021). Comparative study on the novel umami-active peptides of the whole soybeans and the defatted soybeans fermented soy sauce. J. Sci. Food Agric..

[29] Zhu X., Sun-Waterhouse D., Chen J., Cui C., Wang W. (2020). Bitter-tasting hydrophobic peptides prepared from soy sauce using aqueous ethanol solutions influence taste sensation. Int. J. Food Sci. Technol..

[30] Zhao J., Liao S., Bi X., Zhao J., Liu P., Ding W., Che Z., Wang Q., Lin H. (2022). Isolation, identification and characterization of taste peptides from fermented broad bean paste. Food Funct..

[31] Yang H., Zong X., Cui C., Mu L., Zhao H. (2018). Wheat gluten hydrolysates separated by macroporous resins enhance the stress tolerance in brewer’s yeast. Food Chem..

[32] Rhyu M.-R., Kim E.-Y. (2011). Umami taste characteristics of water extract of Doenjang, a Korean soybean paste: Low-molecular acidic peptides may be a possible clue to the taste. Food Chem..

[33] Cheng K., Wang S., Wang Y., Bao Y., Gao P., Lei L., Liang H., Zhang S., Dong L. (2023). Modification of a Novel Umami Octapeptide with Trypsin Hydrolysis Sites via Homology Modeling and Molecular Docking. J. Agric. Food Chem..

[34] Zhang Y., Tang X., Li F., Zhang J., Zhang B., Yang X., Tang Y., Zhang Y., Fan J., Zhang B. (2022). Inhibitory effects of oat peptides on lipolysis: A physicochemical perspective. Food Chem..

[35] Dang Y., Hao L., Cao J., Sun Y., Zeng X., Wu Z., Pan D. (2019). Molecular docking and simulation of the synergistic effect between umami peptides, monosodium glutamate and taste receptor T1R1/T1R3. Food Chem..

[36] Yu Z., Jiang H., Guo R., Yang B., You G., Zhao M., Liu X. (2018). Taste, umami-enhance effect and amino acid sequence of peptides separated from silkworm pupa hydrolysate. Food Res. Int..

[37] Zhao Y., Zhao X., Sun-Waterhouse D., Waterhouse G.I.N., Zhao M., Zhang J., Wang F., Su G. (2021). Two-stage selective enzymatic hydrolysis generates protein hydrolysates rich in Asn-Pro and Ala-His for enhancing taste attributes of soy sauce. Food Chem..

[38] Chen M., Gao X., Pan D., Xu S., Zhang H., Sun Y., He J., Dang Y. (2021). Taste characteristics and umami mechanism of novel umami peptides and umami-enhancing peptides isolated from the hydrolysates of Sanhuang Chicken. Eur. Food Res. Technol..

[39] Oka S., Nagata K. (1974). Isolation and characterization of acidic peptides in soy sauce. Agric. Biol. Chem..

[40] Li X., Xie X., Wang J., Xu Y., Yi S., Zhu W., Mi H., Li T., Li J. (2020). Identification, taste characteristics and molecular docking study of novel umami peptides derived from the aqueous extract of the clam Meretrix meretrix Linnaeus. Food Chem..

[41] Kim M.J., Son H.J., Kim Y., Misaka T., Rhyu M.-R. (2015). Umami–bitter interactions: The suppression of bitterness by umami peptides via human bitter taste receptor. Biochem. Biophys. Res. Commun..

[42] Yang L., Dai B., Ayed C., Liu Y. (2019). Comparing the metabolic profiles of raw and cooked pufferfish (*Takifugu flavidus*) meat by NMR assessment. Food Chem..

[43] Gao B., Hu X., Li R., Zhao Y., Tu Y., Zhao Y. (2021). Screening of characteristic umami substances in preserved egg yolk based on the electronic tongue and UHPLC-MS/MS. LWT.

[44] Wang W., Cui Z., Ning M., Zhou T., Liu Y. (2022). In-silico investigation of umami peptides with receptor T1R1/T1R3 for the discovering potential targets: A combined modeling approach. Biomaterials.

[45] Wang W., Yang L., Ning M., Liu Z., Liu Y. (2022). A rational tool for the umami evaluation of peptides based on multi-techniques. Food Chem..

[46] Zhang L., Pu D., Zhang J., Hao Z., Zhao X., Sun B., Zhang Y. (2023). Identification of novel umami peptides in chicken breast soup through a sensory-guided approach and molecular docking to the T1R1/T1R3 taste receptor. J. Agric. Food Chem..

[47] Liang L., Zhou C., Zhang J., Huang Y., Zhao J., Sun B., Zhang Y. (2022). Characteristics of umami peptides identified from porcine bone soup and molecular docking to the taste receptor T1R1/T1R3. Food Chem..

[48] Zhang J., Su G., Zhao T., Fan J., Ho C.-T., Zhao M. (2022). Preparation, Sensory Characterization, and Umami-Enhancing Mechanism of Novel Peptide Glycoconjugates. J. Agric. Food Chem..

[49] Chakraborty D., Chattaraj P.K. (2021). Conceptual density functional theory based electronic structure principles. Chem. Sci..

[50] Shi C., Liu M., Zhao H., Lv Z., Liang L., Zhang B. (2022). A novel insight into screening for antioxidant peptides from hazelnut protein: Based on the properties of amino acid residues. Antioxidants.

[51] Yu X., Zhang L., Miao X., Li Y., Liu Y. (2017). The structure features of umami hexapeptides for the T1R1/T1R3 receptor. Food Chem..

