# Supplementary material for: Sensory-Guided Isolation, Identification, and Active Site Calculation of Novel Umami Peptides from Ethanol Precipitation Fractions of Fermented Grain Wine (Huangjiu)
Source: Foods. 2023 Sep 11;12(18):3398. doi: 10.3390/foods12183398 (PMC10527695; doi:10.3390/foods12183398)

Supplementary Tables and Figures

Table S1. Umami prediction and docking screening results of 75%ETP water eluted fraction peptides by macroporous resin

Serial	Peptide Sequence	Affinity (kca/mol) T1R1	Affinity (kca/mol) T1R3	UMPred-FRL Score	UmamiDB Score	Intensity	Mass (Da)
1	DTYNPR	-11.0	-10.1	0.641	0.991	49703000	750.33
2	SYNPR	-10.6	-9.1	0.949	0.877	148890000	635.30
3	TYNPR	-10.8	-9.5	0.984	0.983	109380000	649.31
4	FRDEHQ	-10.0	-9.9	0.603	0.982	268490000	830.37
5	TYNPK	-10.0	-8.9	0.572	0.912	6808700	621.31
6	RFRQGD	-9.9	-8.9	0.800	0.985	8428200	777.39
7	KHHDRPG	-9.8	-9.4	0.874	0.934	338970	845.42
8	NFHHGD	-9.7	-9.7	0.866	0.895	307450000	725.29
9	ENFHHGD	-9.6	-10.0	0.618	0.896	3848100	854.33
10	FHHGD	-9.6	-9.1	0.935	0.759	93945000	611.24
11	FRDEH	-9.6	-9.9	0.578	0.843	485570000	702.31
12	FHTGDRG	-9.3	-10.0	0.809	0.829	3713000	788.35
13	LTESQSQSH	-9.1	-9.4	0.507	0.995	779040	1015.45
14	TVDGPSH	-9.1	-9.9	0.545	0.768	23113000	711.32
15	VVGVNH	-9.1	-9.0	0.737	0.504	1225500	623.34
16	LAGNKRNP	-8.5	-9.3	0.814	0.972	5046900	868.49
17	EGLERELEK	-8.2	-8.4	0.879	0.604	1881500	1101.56
18	KDEH	-8.2	-7.8	0.947	0.986	1351800	802.40
19	KKRGDTKDH	-7.8	-9.0	0.960	0.986	31852	1083.58
20	DNSSTISTHD	-7.7	-8.7	0.916	0.609	875960	1075.44
21	SIEQHSSQN	-7.4	-9.5	0.596	0.609	6691900	1028.45
22	SSIEQHSSQN	-7.4	-9.2	0.717	0.609	1225300	1115.48

Figure S1. Ramachandran plot of the moldeled umami receptor T1R1/T1R3

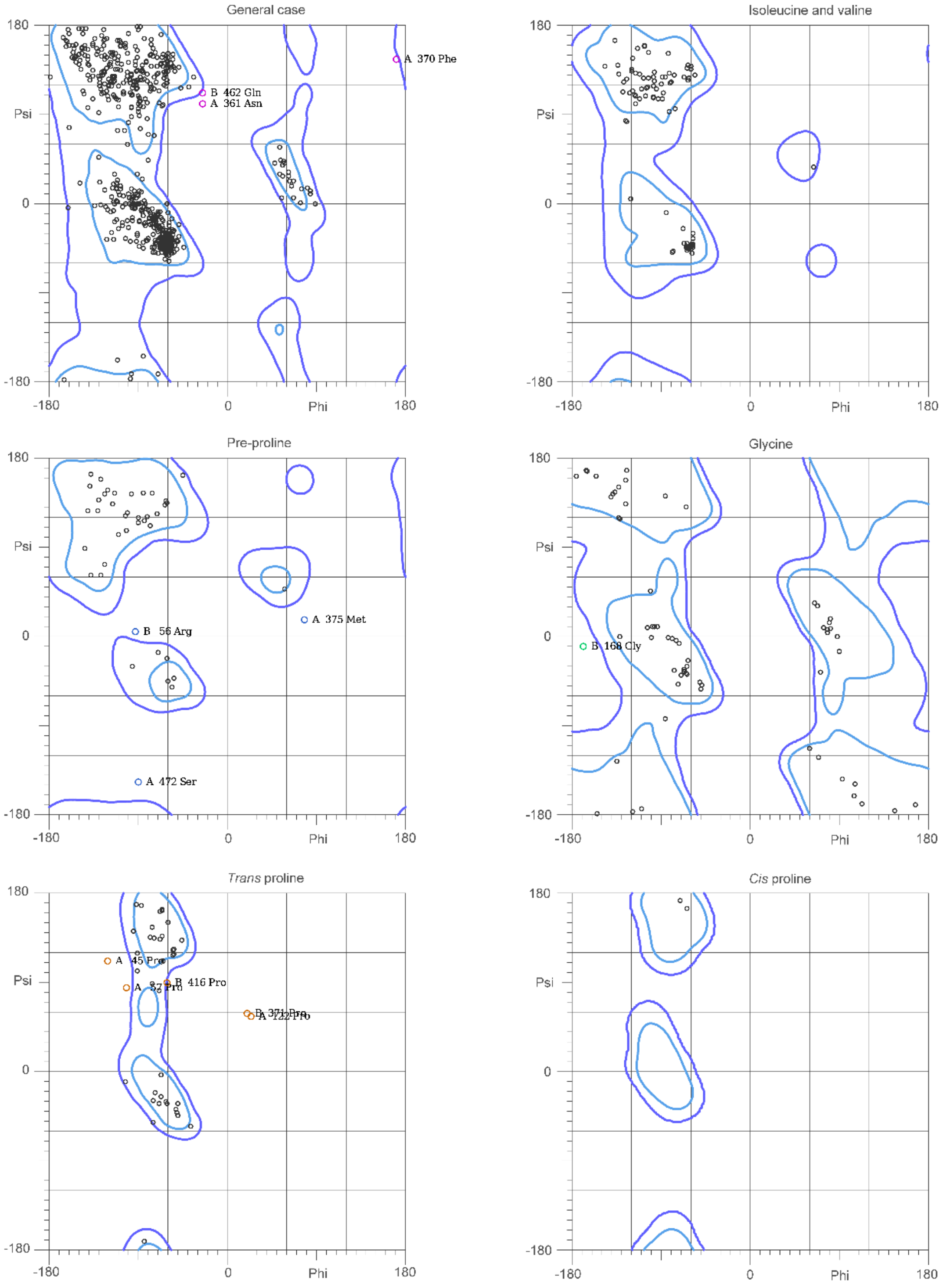

Figure S2. The docking residues interaction mode between umami peptides and T1R1-T1R3 receptor.

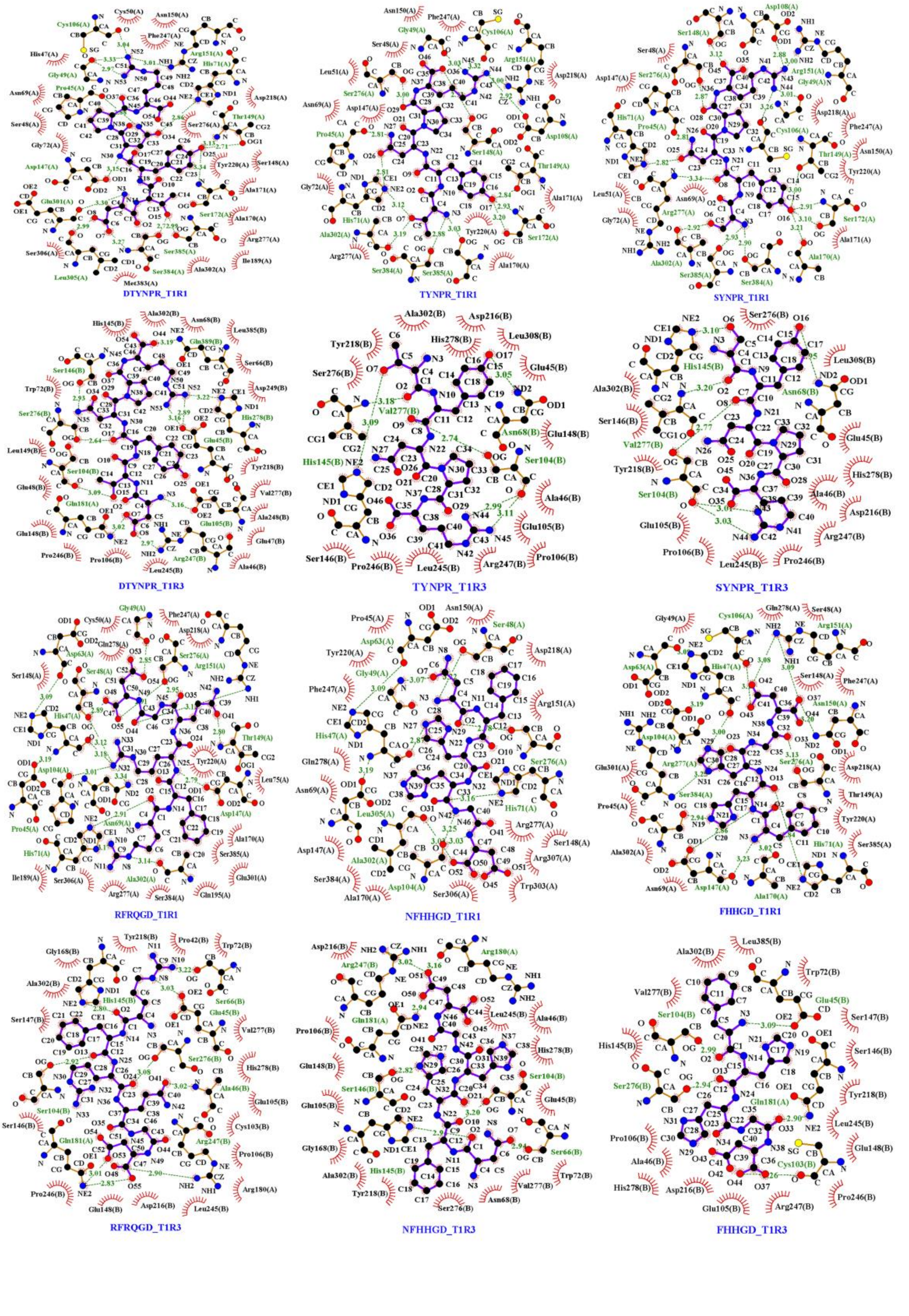

Supplement: Supplementary file 1 [file foods-12-03398-s001.zip › foods-2607463-supplementary.pdf]
